# Epidemiological Profile and Awareness Regarding Animal Bite Management in an Urban Area

**DOI:** 10.7759/cureus.69159

**Published:** 2024-09-11

**Authors:** Sushma Mallikarjuna, Merlin Shruthi Jeshanah

**Affiliations:** 1 Community Medicine, St. Peter's Medical College, Hosur, IND

**Keywords:** animal bite management, bite wound care, post-exposure prophylaxis (pep), rabies awareness, urban animal bites

## Abstract

Objectives: The most common way for people to contract the deadly zoonotic disease rabies is through animal bites. This study was designed because there is a shortage of community-based data to determine the true extent of rabies infection and a lack of understanding regarding patient misconceptions in urban areas. The objective was to determine the scope of the issue and the epidemiological features of animal bite incidents.

Methodology: This was a facility-based cross-sectional study conducted at the field practice area (Urban Health Training Centre) under the Department of Community Medicine, Government Medical College. The study duration was one year (May 2023 to April 2024). Consecutive patients (accompanied by an attendant) attending the Urban Health Training Centre were included in the study. A purposive sampling technique was used, and around 400 animal bite cases were recorded in one year at the Urban Health Training Centre.

Results: Out of 1503 male cases at the Urban Health Training Centre, around 255 were dog bite cases (50.6%), whereas out of 1302 females visiting the Urban Health Training Centre, around 145 were dog bite cases (48%). The majority (35%) belonged to the age group of 11 to 20 years for both males and females. The mean age of the cases was 19.4 ± 3.4 years. The study found a male preponderance (64%), with females constituting 36%. The right lower limb (49.5%), left lower limb (39%), and upper limb were the most frequently bitten sites. Fifty-one percent of the cases fell into Category II. According to local data, the most popular home management practices were ghee oil and cold treatments. Although Category III is the most severe bite, only 35% had taken the anti-rabies vaccine (ARV), and 8.75% had received rabies immunoglobulin.

Conclusion: The public and medical community need to be made aware of the importance of wound care and the prudent use of the anti-rabies vaccine.

## Introduction

Animal bites, particularly those from dogs and cats, represent a significant public health concern in urban environments worldwide. These incidents pose risks not only due to immediate physical injuries but also due to the potential transmission of zoonotic diseases, most notably rabies. Effective management and prevention strategies are essential to mitigate these risks and ensure public safety [1].

The history of rabies dates back to ancient Egypt. The most common way for people to contract this deadly zoonotic disease is through animal bites. Rabies primarily affects terrestrial and flying creatures, including humans, dogs, wolves, foxes, coyotes, jackals, cats, bobcats, lions, mongooses, skunks, badgers, bats, and monkeys. In India, dogs are the primary carriers of rabies. Researchers estimate that rabid dogs in Africa and Asia cause approximately 55,000 deaths from rabies each year [2].

Understanding the epidemiological profile of animal bites involves analyzing the incidence, demographic distribution, and outcomes of bite cases. Age, gender, socioeconomic status, and geographic location within urban settings can influence the likelihood of animal bites. Moreover, the types of animals involved, the nature of interactions leading to bites, and seasonal variations are critical to consider. Data from healthcare facilities, veterinary services, and public health records are instrumental in mapping these epidemiological patterns [3,4].

In India, where rabies is prevalent, dogs account for most animal bites (91.5%). Of these incidents, approximately 60% involve stray animals, while 40% involve pets. In 2005, approximately 12,700 people in India died from clearly identifiable furious rabies, equivalent to 1.1 deaths per 1,000 people. The majority of these deaths (62%) occurred in rural areas, with 91% affecting children under the age of 15. Uttar Pradesh accounted for one-third of these deaths. In contrast, seven central and southeastern states (Chhattisgarh, Uttar Pradesh, Orissa, Andhra Pradesh, Bihar, Assam, and Madhya Pradesh) accounted for three-quarters of these deaths [1,2].

Awareness regarding animal bite management encompasses knowledge of first aid, post-exposure prophylaxis (PEP), and timely medical intervention. It also involves understanding preventive measures, such as responsible pet ownership, vaccination of pets, and public education on avoiding risky animal interactions. Evaluating the level of awareness among different population groups can identify gaps in knowledge and highlight areas in need of targeted educational campaigns [5-7].

This study aims to provide a comprehensive overview of the epidemiological characteristics of animal bites in an urban area and assess awareness levels regarding their management. It seeks to inform public health strategies and policies that can enhance prevention, provide timely treatment, and ultimately reduce the incidence and impact of animal bites. Additionally, the findings can guide the development of community-based interventions and education programs to foster a safer coexistence between humans and animals in urban settings.

Understanding the epidemiological profile and raising awareness about animal bite management is crucial for devising effective public health measures and improving the overall health and safety of urban populations.

## Materials and methods

The Department of Community Medicine at Government Medical College conducted a facility-based cross-sectional study at the Urban Health Training Center (UHTC) field practice area from May 2023 to April 2024.

Inclusion criteria encompassed consecutive patients who were accompanying attendees at UHTC. A purposive sampling technique was employed, recording approximately 400 animal bite cases within one year. Individuals with a history of previous animal bites were excluded. The Institutional Ethics Committee approved the study before commencement.

Procedure

A pretested, predesigned questionnaire with an interview schedule was utilized. This instrument underwent preliminary translation, back translation, and retranslation, followed by a pilot study to ensure validity. The questionnaire covered demographic information, family features (such as residency and type), management style, common misconceptions, and the need for immediate treatment.

Participants who refused to participate were excluded. The “Informed Consent Form” was translated from English to Hindi with assistance from faculty members and specialists in animal bites. A bite was considered provoked if the subject initiated interaction with the dog, such as playing with it or disturbing it while it was eating. The recall period for incidents was one year.

The study area comprised a neighborhood with a population of 20,000. Five surveyors were selected from five randomly chosen UHTC pockets, and paramedical staff received rabies training from an expert through briefing and debriefing sessions under the investigators’ supervision. Health providers informed families about the study and, if necessary, the extent of their future involvement while encouraging their participation.

Each participant was informed about the study’s objectives. All participants managed the process well, with no medical complaints reported. Data collection, including counseling sessions, took less than ten minutes.

Statistical analysis

Data were entered into the Statistical Package for the Social Sciences (SPSS) Version 25 (IBM Corp., Armonk, NY, USA) for analysis. Categorical variables were expressed as frequencies and percentages, while continuous variables were reported as means and standard deviations. The frequency, proportion, and mean of the data were analyzed. The chi-square test was used to compare categorical data, with statistical significance set at p < 0.05.

## Results

According to Table 1, out of 1,503 male cases at UHTC, approximately 255 were dog bite cases (50.6%). Among 1,302 female visitors to UHTC, around 145 were dog bite cases (nearly 48%). The majority (35%) of these cases were in the 11 to 20 years age group, for both males and females. The mean age of the cases was 19.4 ± 3.4 years. The study found a male preponderance (64%) compared to females (36%). The overall incidence of dog bite cases was higher in the adolescent age group, likely because they spend more time outside the home. Fewer cases were reported in individuals older than 40 years, and this difference was not statistically significant (p > 0.05).

**Table 1:** Gender- and age-wise distribution of study participants (N=400)

Age groups (years)	Male		Female		p-value
	N	Dog bite cases	N	Dog bite cases	0.07
<10 years	237	15	115	10	
11 to 20	460	90	495	50	
21 to 30	346	80	345	39	
31 to 40	275	40	250	30	
41 to 50	160	20	65	10	
>50 years	25	10	32	6	

According to Table 2, the most frequently bitten sites were the right lower limb (49.5%), left lower limb (39%), and upper limbs (percentage not provided). Fifty-one percent of the cases fell into Group II. The most common local management method, according to local data, was the application of ghee oil, followed by cold treatment. This local application served as pre-treatment. Just over 37.5% of patients had not recovered fully. Over 45% of cases were reported between 24 and 48 hours after the bite, and 39% were reported after two days. The majority of cases (54%) received treatment from government facilities, reflecting a strong understanding of the cost-effectiveness of immunizations.

**Table 2:** Distribution of participants as per site of bite, health-seeking behavior, and category

Characteristics	Dog bite cases (N=400)
Site of Bite	
Upper limb	28 (7%)
Trunk	10 (2.5%)
Head and Neck	8 (2%)
Right lower limb	198 (49.5%)
Left lower limb	156 (39%)
Category	
I	20 (5%)
II	203 (51%)
III	177 (44%)
Pattern of House Management	
Chili	80 (20%)
Oil	60 (15%)
Turmeric Powder	30 (7.5%)
Only water	54 (13.5%)
Soap and Water	24 (6%)
No management	150 (37.5%)
Time of Reporting	
<24 hours	65 (16%)
24 to 48 hours	180 (45%)
>48 hours	155 (39%)
Treatment Facility	
Private hospital	183 (46%)
Government Hospital	217 (54%)

According to Table 3, around 80% of cases were related to the menace of stray dogs. When asked what should be done after a dog bite in areas with stray dog issues, 32.5% suggested monitoring the dog for 10 days, while 35% recommended letting the dog go. This difference was found to be statistically significant (p < 0.05).

**Table 3:** Awareness of study participants towards dog bite

Menace of stray dogs	Watch the dog (10 days)	Kill the dog	Let the dog go away	p-value
Yes	134 (33.5%)	51 (12.7%)	140 (35%)	0.01*
No	36 (9%)	21 (5.25%)	18 (4.5%)	

As per Table 4, most cases were categorized as Category II. Although Category III represents the most severe bites, only 35% of patients received post-exposure anti-rabies vaccination (ARV) and 8.75% received rabies immunoglobulin (RIG). This low uptake may be due to the high cost and limited availability of RIG, which may have led patients to forgo it. This difference was found to be statistically significant (p < 0.05).

**Table 4:** Association of anti-rabies management with category of bite ARV: anti-rabies vaccine, RIG: rabies immunoglobulin

Category	ARV with RIG				p-value
	Male		Female		0.03*
	ARV	RIG	ARV	RIG	
I	13	0	7	0	
II	145	7	46	5	
III	71	19	70	17	

## Discussion

Our study observed that dogs were the primary source of bites, responsible for 50.6% of the cases among males and 48% among females. This finding is lower compared to studies by Gujalwar et al. [8] and Sudarshan et al. [9], which reported higher percentages of dog bites. The discrepancy could be attributed to variations in regional animal populations and human-animal interactions. Similar to other studies, adolescents (11-20 years) were the most affected group, possibly due to their increased outdoor activities consistent with findings from Trivedi et al. [10] and Meshram et al. [11], where younger age groups showed higher vulnerability to bites. Our study noted a mean age of 19.4 ± 3.4 years, which aligns with the demographic trends seen in similar research.

Stray dogs inflicted the majority of bites in our study (80%), a significant issue also highlighted by Acharya et al. [12] and Krishna et al [13]. Our findings underscore the public health challenge posed by stray animals, as reflected by the 35% of participants who suggested letting the dog go after a bite incident despite the associated health risks. This attitude towards stray dogs differs from studies like Shah et al. [14], who reported a higher percentage of stray dog involvement (96.2%) and a stronger emphasis on regulating stray animal populations.

The lower limb was the most common site of bite in our study (49.5% right lower limb and 39% left lower limb), which is consistent with studies by Masoodi et al. [15], Nimalae et al. [16], and Gujalwar et al. [8]. Bites to the lower limbs are common because they are the most accessible part of the body for animals, especially dogs. Although upper limb bites were less frequent (7%), they were more commonly seen in children, supporting findings from similar studies.

A concerning aspect of our study was the low rate of proper wound management. Only 6% of cases used soap and water for wound cleansing, which contrasts sharply with higher rates reported by Jana et al. [17] and Krishna et al. [13], where over 60% of patients practiced proper wound care. Instead, traditional methods such as applying ghee oil (15%) and cold treatment (20%) were common. These alternative treatments reflect a lack of awareness about proper wound care, as also seen in the study by Sharma et al. [18], who similarly reported low rates of soap and water usage.

In terms of medical treatment, 97.7% of our cases received the ARV. Still, only 8.75% received RIG, even among Category III cases, which reflects a severe gap in treatment, consistent with findings from Sukhsohale et al. [19] and Gogtay et al. [20], who reported poor use of life-saving RIG. The low uptake of RIG in our study can be attributed to its high cost and limited availability, mirroring the challenges highlighted by Pattanayak et al. [21] and Sahu et al. [22] in their respective regions. Our data underscore the need for improving access to RIG and raising public awareness about its importance in managing severe bites.

Limitations of the study

This study has several notable limitations that must be acknowledged. First, the cross-sectional design of the survey inherently limits the ability to establish causality. While the study provides a snapshot of the prevalence and characteristics of dog bites, it does not allow for conclusions about cause-and-effect relationships between the examined factors and the observed outcomes. Second, the study was conducted in a specific urban area, which may not be representative of other geographical locations, particularly rural areas. In rural settings, the frequency of dog bites and the management practices might differ significantly due to variations in animal control, healthcare access, and public awareness. Consequently, the findings may not be generalizable beyond the urban context in which the study was performed.

Additionally, the reliance on self-reported data introduces the potential for recall bias. Participants’ recollections about how they managed their bites and the timing of their treatment may not always be accurate, which could affect the reliability of the information provided. For instance, participants may forget details about the application of traditional therapies or may misreport the exact timing of their medical consultations. These factors can lead to inaccuracies in the data and potentially skew the study’s findings. Therefore, while the study offers valuable insights, its limitations should be considered when interpreting the results and applying them to broader contexts.

## Conclusions

In conclusion, this study demonstrates a significant prevalence of dog bites among adolescents, particularly males, with the lower limbs being the most commonly affected area. Despite the moderate severity of most cases (Category II), there remains a notable gap in the uptake of essential post-exposure prevention, such as ARV and RIG, primarily due to financial and accessibility barriers. Furthermore, the reliance on traditional treatments highlights the need for comprehensive public education on proper wound care and timely medical intervention. To improve health outcomes related to dog bites, it is crucial to enhance public awareness campaigns and increase access to rabies prevention, particularly in regions with high stray dog populations.

## Appendices

Questionnaire

Section A: Demographic Information

1. Age:

  - Under 18

  - 18-30

  - 31-45

  - 46-60

  - Above 60

2. Gender:

  - Male

  - Female

  - Other

3. Education Level:

  - No formal education

  - Primary school

  - Secondary school

  - College/University

  - Post-graduate

4. Occupation:

  - Student

  - Employed

  - Self-employed

  - Unemployed

  - Retired

  - Other (please specify)

5. Area of Residence:

  - Urban slum

  - Urban non-slum

  - Semi-urban

  - Other (please specify)

6. Do you own a pet?

  - Yes

  - No

Section B: Experience with Animal Bites

7. Have you or anyone in your family ever been bitten by an animal?

  - Yes

  - No

8. If yes, what type of animal caused the bite?

  - Dog

  - Cat

  - Monkey

  - Rodent

  - Other (please specify)

9. Where did the bite occur?

  - Home

  - Street

  - Park

  - Workplace

  - Other (please specify)

10. What part of the body was affected?

   - Hands

   - Legs

   - Face/Head

   - Other (please specify)

11. Did the bite result in any medical complications?

   - Yes

   - No

Section C: Awareness and Knowledge about Animal Bite Management

12. Are you aware of the risks associated with animal bites (e.g., rabies)?

   - Yes

   - No

13. Do you know what immediate steps should be taken after an animal bite?

   - Yes

   - No

14. If yes, what actions would you take immediately after an animal bite?

   - Wash the wound with soap and water

   - Apply antiseptic

   - Visit a healthcare facility

   - Do nothing

   - Other (please specify)

15. Are you aware of the vaccination protocol for rabies post-animal bite?

   - Yes

   - No

16. Where would you seek medical assistance in case of an animal bite?

   - Government hospital/clinic

   - Private hospital/clinic

   - Pharmacy

   - Traditional healer

   - Other (please specify)

17. Have you received information or education on animal bite management from any of the following sources? (Select all that apply)

   - Healthcare providers

   - Schools/Colleges

   - Media (TV, radio, newspaper)

   - Community programs

   - Social media

   - Other (please specify)

18. Do you believe there is adequate awareness regarding animal bite management in your community?

   - Yes

   - No

Section D: Prevention and Control Measures

19. Do you think there should be stricter regulations for pet ownership in urban areas to prevent animal bites?

   - Yes

   - No

20. Are you aware of any community-based animal control programs (e.g., stray dog management)?

   - Yes

   - No

21. What preventive measures do you think should be implemented to reduce animal bites in urban areas? (Select all that apply)

   - Stricter pet ownership laws

   - Public education campaigns

   - Vaccination programs for animals

   - Stray animal control

   - Other (please specify)

22. Do you believe that healthcare facilities in your area are well-equipped to manage animal bite cases?

   - Yes

   - No

23. Would you participate in an awareness campaign on animal bite prevention and management?

   - Yes

   - No
